# The Antifouling and Drag-Reduction Performance of Alumina Reinforced Polydimethylsiloxane Coatings Containing Phenylmethylsilicone Oil

**DOI:** 10.3390/polym13183067

**Published:** 2021-09-10

**Authors:** Qiang Yang, Zhanping Zhang, Yuhong Qi, Hongyang Zhang

**Affiliations:** Department of Materials Science and Engineering, Dalian Maritime University, Dalian 116026, China; 1120180341yq@dlmu.edu.cn (Q.Y.); yuhong_qi@dlmu.edu.cn (Y.Q.); zhy686@dlmu.edu.cn (H.Z.)

**Keywords:** polydimethylsiloxane, alumina, elastic modulus, anti-fouling, drag-reduction

## Abstract

Fouling-release coatings reinforced with micro-alumina and nano-alumina were prepared based on polydimethylsiloxane (PDMS) containing phenylmethylsilicone oil. The surface properties, mechanical properties, leaching behavior of silicone oil, anti-fouling and drag-reduction performance of the coating were studied. The results show that the addition of alumina can significantly improve the tensile strength, elastic modulus and Shore’s hardness of the coating. The adhesion experiments of marine bacteria and *Navicula Tenera* show that the addition of alumina can reduce the antifouling performance of the coating, which is related to the stripping mode of fouling organisms. The fouling organisms leave the coating surface by shearing, and the energy required for shearing is proportional to the elastic modulus of the coating. At 800–1400 rpm, the addition of alumina will reduce the drag reduction performance of the coating, which is related to the drag reduction mechanism of PDMS. PDMS counteracts part of the resistance by surface deformation. The larger the elastic modulus is, the more difficult the surface deformation is. The experiment of silicone oil leaching shows that the increase of alumina addition amount and the decrease of particle size will inhibit the leaching of silicone oil.

## 1. Introduction

The attachment of marine life to the hull surface will increase the navigation resistance and the weight of the ship, resulting in the reduction of the ship’s speed [1,2]. To maintain a high speed, high fuel consumption is necessary, which leads to environmental damage and energy consumption [3]. Therefore, measures are needed to hinder the fouling process of marine life. The most effective way is to use anti-fouling coatings. Traditional anti-fouling coatings are based on the release of toxic substances to achieve the purpose of anti-fouling and drag-reduction [4,5]. Tributyltin has a broad spectrum of antibacterial effect. However, the tributyltin compounds are aggregated in the organism through the food chain, causing organ lesions and deformity [6]. Therefore, it is disabled by the international maritime organization.

Polydimethylsiloxane (PDMS) coating is an environmentally friendly coating with low surface energy and elastic modulus [7,8]. These properties can reduce adhesion between PDMS and marine organisms to keep the surface smooth [9]. However, the mechanical properties of PDMS are poor, which is related to its molecular chains [10].The Si–O chain of PDMS is different from traditional –C–C– chains, with greater flexibility and malleability.

At present, researchers have made many experiments to improve the mechanical properties of the silicone coatings. Common modified methods are modified to PDMS materials and the reinforcement of powder [11,12,13,14,15]. Modified PDMS materials are based on PDMS, and other resins provide high mechanical properties. Zhao modified PDMS with γ-aminopropyltriethoxysilane and epoxy resin as raw materials [12]. The results show that the introduction of epoxy resin can successfully enhance the mechanical properties of the coatings. However, the compatibility of PDMS with other materials is poor, and phase separation will occur under certain conditions, which will affect the performance of materials [13].

As an important part of the coatings, powder not only affects the coloring and covering, but also affects the mechanical properties of the coatings [14,16]. The molecular structure of gas phase silica is similar to that of silicone resin, and the addition of gas phase silica to the coatings can obviously enhance the mechanical properties of the coatings [17]. Liu et al. [18] prepared organic–inorganic composite coatings using fluorinated silica particles as raw materials. The coating shows good mechanical properties. Ba et al. [19] add nano titanium dioxide to PDMS by mechanical stirring. The experimental results show that the addition of nano titanium dioxide can significantly improve the mechanical properties of the coatings. Yang et al. [20] studied the effect of the amount of barium sulfate on the mechanical properties of the resin. The results show that BaSO_4_ particles can enhance the impact toughness and fracture elongation of the resin at the same time. The leaching of silicone oil from PDMS coatings can improve the anti-fouling and drag-reduction performance of the coatings [21].

Alumina is a kind of hard powder with high hardness and a strong wear-resisting property. In this paper, alumina reinforced PDMS coatings containing phenylmethylsilicone oil (PSO) were prepared by two-step method. The effects of alumina addition amount and particle size on the mechanical properties, surface properties, anti-fouling and drag-reduction properties of the coatings were studied.

## 2. Materials and Methods

### 2.1. Experimental Materials and Equipment

Hydroxyl-terminated polydimethylsiloxane (PDMS, 31900-57-9) with the viscosity of 10,000 mPa·s was used as the key film forming material, which was purchased from Dayi Chemical Industry Co. Ltd. (Yantai, China). Tetraethylorthosilicate (TEOS, 68412-37-3, analytical grade), used as crosslinking curing agent, and Dibutyltin dilaurate (DBTDL, 77-58-7, analytical grade), were produced by Kemiou Chemical Reagent Co., Ltd. (Tianjin, China). Non-reactive PSO with a viscosity of 30 mPa·s was obtained from Hualing Resin Co., Ltd. (Shanghai, China). *Navicula Tenera* was cultured by the Qingdao Chinese Academy of Sciences Seaweed Germplasm Bank (Qingdao, China). Micro-alumina and nano-alumina were produced by Beijing Nachen Technology Development Co., Ltd. (Beijing, China). All powders were dried at 120 °C for 24 h before the experiment.

### 2.2. Preparation of Al_2_O_3_ Reinforced Coatings

#### 2.2.1. Al_2_O_3_ Reinforced PDMS Topcoat

**Figure 1:**
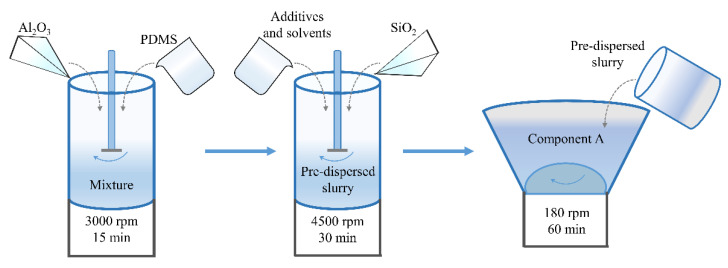
Schematic diagram of preparation process for component A of silicone topcoat.

Preparation of Al_2_O_3_ reinforced PDMS coatings by two-step method. In order to directly find the effect of alumina on the properties of the coatings, only fumed silica and alumina were used as pigments and fillers. Firstly, each PDMS pre-slurry with different alumina content and particle size were prepared by mechanical stirring at 3000 rpm for 15 min with BGD 750 Versatile Sand-Milling Dispersion-agitator (Guangzhou Biuged Laboratory Instrument Supplies Co., Ltd., Guangzhou, China). Then, PSO, additives and fumed silica were added to the slurry, and stirred at 4500 rpm for 30 min. After that, the mixed liquid was poured into cone mill and ground for 60 min, then put it into a tinplate container for use as the paint component A. The specific process was shown in Figure 1. As the curing agent of the paint, TEOS was marked as component B. DBTDL was labeled as component C as catalyst. The three components A, B, and C were mixed at 25:4:1. According to different painting requirements, a certain amount of xylene can be added to adjust its viscosity.

The paint was sprayed on the glass slide with a dimension of 75 mm × 25 mm × 1 mm to measure the drag reduction and surface properties of the coating. Pour the paint into a Teflon Mold with a dimension of 150 mm × 150 mm × 2 mm and measure the mechanical properties of the coating.

According to the addition and particle size of alumina to mark, the composition of the component A is shown in Table 1. MA50 and NA30 in X-Y represent the particle size of alumina, respectively, where MA50 represents the particle size of alumina as 30–50 μm, and NA30 represents the particle size of alumina as 30 nm. The “Y” in X-Y represents the ratio of the mass of alumina and component A, multiplied by 100.

#### 2.2.2. Preparation of Composite Coatings

**Figure 2:**
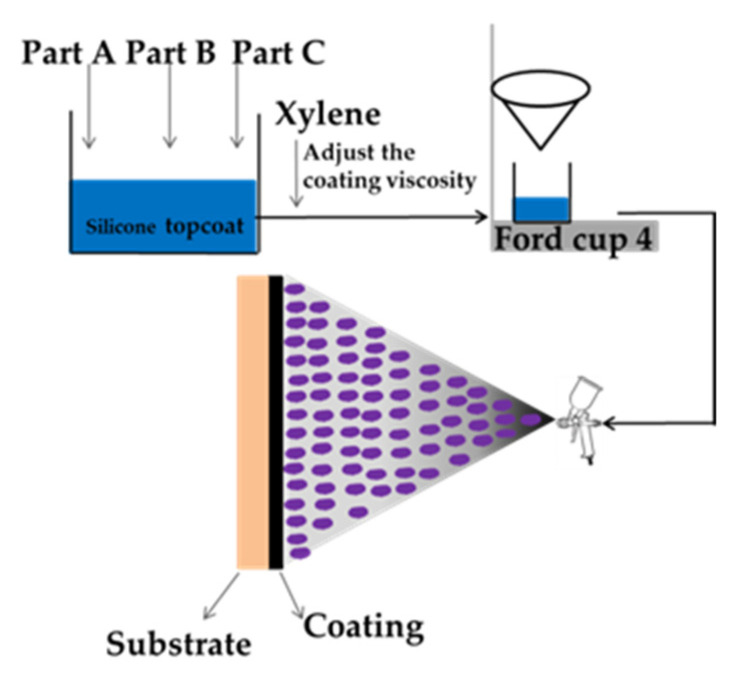
Spraying process of silicone topcoat.

**Figure 3:**
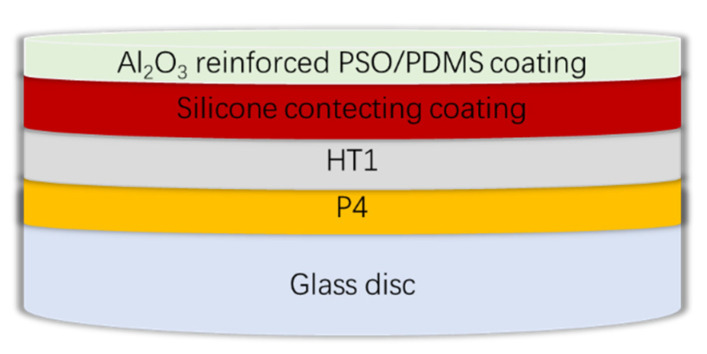
Schematic diagram of composite coatings.

WS T-207 epoxy strontium yellow paint (P4) and WS T-208 SM epoxy paint (HT1) were used as epoxy primer and anti-corrosive coating; they were produced by the 53rd Research Institute of China North Industries Group (Jinan, China). The silicone connecting paint was prepared by Liu [22]. The spraying process is shown in Figure 2. The specific coatings distribution of the composite coatings is shown in Figure 3.

### 2.3. Experiment and Characterization

#### 2.3.1. Contact Angle and Surface Free Energy

Pick five points randomly on the surface of the coating. Use the JC2000 contact angle measuring instrument (Shanghai Zhongchen Digital Technic Apparatus Co., Ltd., Shanghai, China) to measure the water contact angle (WCA) and diiodomethane contact angle (DCA). Based on the measured WCA and DCA, the surface free energy of each coating was calculated by the Owens two-liquid method [23].

#### 2.3.2. Tensile Performance Test

According to Chinese national standard GB/T 528-2009 (IS037-2005), the coating was cut into a 4 mm × 45 mm dumbbell-shaped tensile specimen. The mechanical properties of the coating were measured by the UTM5105 computer-controlled electronic universal testing machine (Jinan Wance Electrical Equipment Co., Ltd., Jinan, China), and the tensile rate was 50 mm/min. The elastic modulus, tensile strength and fracture elongation of the coating was calculated by tensile data.

#### 2.3.3. Crosslinking Density Test

The relative molecular weight (Mc) of the polymer was determined by swelling method. Three 1 cm^2^ coated samples were cut from the same sample film and placed in the AB204-S precision electronic balance (Mettler Toledo Co., Ltd., Zurich, Switzerland). The density of the coatings was measured by MH-300A densitometer (Kunshan Creator Testing Instrument Co., Ltd., Kunshan, China). The sample was put into a centrifuge tube filled with 50 mL toluene in a 25 °C water bath environment. The coatings were taken out every 3 h and the quality of the coatings was weighed in a precision balance. Until the mass difference between two adjacent weightings was less than 0.1 mg, the coating was considered to have reached swelling equilibrium. The sample was placed in a vacuum drying bottle for a period until the mass did not change, and the measured mass was reported as the final swelling equilibrium mass of the coatings. The Mc of the coatings was calculated by Flory–Rhener relation.

#### 2.3.4. Morphology and Roughness

The fast-scanning mode of a confocal laser scanning microscope (CLSM) (Olympus OLS4000, OLYMPUS (China) Co., Ltd., Beijing, China) was used to measure the surface roughness and fracture morphology of the coating. The visual field area of CLSM image is 1282 μm × 1281 μm.

The alumina reinforced PSO/PDMS coatings were sprayed on the glass slide. The surface of the coatings was regularly observed by CLSM and the coverage of PSO leached was calculated by Photoshop software (version 2020). 

#### 2.3.5. Bacteria Attachment Test

The antifouling performance of the coating was verified by bacterial membrane adhesion experiment. The coating was divided into group A and group B, which were immersed in fresh sea water, respectively. After soaking for 48 h, group A was rinsed to simulate the bacterial adhesion of the ship hull in a stationary state. Group B was washed to simulate the bacterial adhesion of the hull during movement. The bacteria were inoculated in 2216E solid medium with a liquid pipette. The Image Pro Plus software was used to calculate the number of bacteria in the solid medium. The 2216E solid culture medium was composed of 2 g peptone, 0.4 g yeast extract, 0.004 g FePO_4_, 8 g agar and 400 mL sterilized seawater, and its detail preparation process can be found in paper [24]. 

#### 2.3.6. *Navicula Tenera* Attachment Test

The antifouling performance of the coating was verified by *Navicula Tenera* adhesion experiment. The coating was divided into two groups: group A was the rinsing sample and group B was the wash sample. The coating was put into sterilized seawater containing *Navicula Tenera* and soaked for 48 h. An ultraviolet–visible spectrophotometer (Labtech UV-2000, Labtech Co., Ltd., Beijing, China) was used to measure the content of chlorophyll-a on the surface of the coating. The attachment and detection methods of *Navicula Tenera* are shown in the literature [25].

#### 2.3.7. Drag Reduction Test

According to Chinese national standard GB/T 7791-2014, drag reduction was tested [26]. A water tank (height 0.62 m, radius 0.3 m) containing 175 L natural sea water was needed for this experiment. When the coating sample (radius 0.05 m) was fixed on the rotating shaft and placed in natural sea water, it was necessary to ensure that the height of the upper surface of the coating was less than 0.3 m of the sea water height. The rotating speed was respectively carried out at 800 rpm, 1000 rpm, 1200 rpm, 1400 rpm, 1600 rpm and 1800 rpm, and the corresponding simulated speed was successively 8.14 knots, 10.18 knots, 12.21 knots, 14.25 knots, 16.28 knots and 18.32 knots. 

## 3. Results and Discussion

### 3.1. Surface Properties

**Figure 4:**
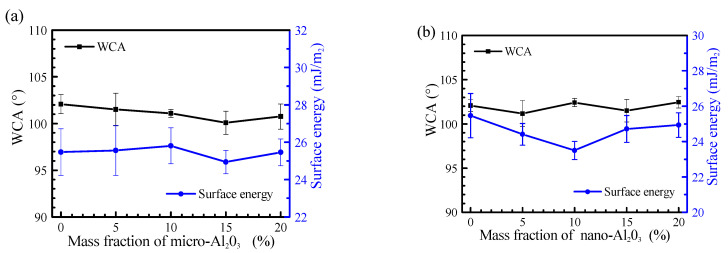
Effect of addition amount and particle size of alumina on WCA and surface energy: (**a**) Micro-alumina, (**b**) Nano-alumina.

The influence of alumina on the surface properties is shown in Figure 4. The addition and particle size of alumina have little effect on the surface energy and WCA of the coatings. Because the addition of alumina does not reach the dispersion limit of silicone resin, it cannot produce a certain size of micro-nanostructure on the surface of the coatings. The alumina used is lipophilic, which can ensure its uniform distribution in the coatings. This was also confirmed by surface roughness of the coatings by using CLSM; the surface roughness of each coating sample had almost no obvious difference. The increase of alumina content and the change of particle size will not have a great effect on the surface roughness of the coatings: for example, the roughness of the coating A-0, MA50-5, MA50-15, NA30-5 and NA30-15 is 0.21 ± 0.03 μm, 0.19 ± 0.01 μm, 0.13 ± 0.01 μm, 0.13 ± 0.07 μm and 0.14 ± 0.03 μm.

### 3.2. Observation of Leached PSO

**Figure 5:**
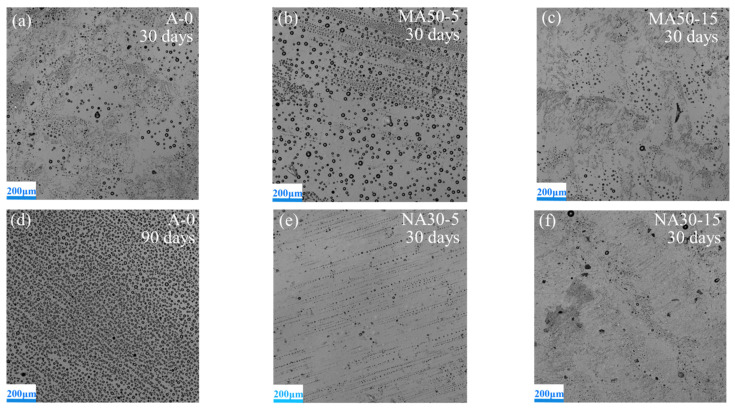
CLSM images of the coatings after exposure in air: (**a**) A-0 30 days, (**b**) MA50-5 30 days, (**c**) MA50-15 30 days, (**d**) A-0 90 days, (**e**) NA30-5 30 days, (**f**) NA30-15 30 days.

**Figure 6:**
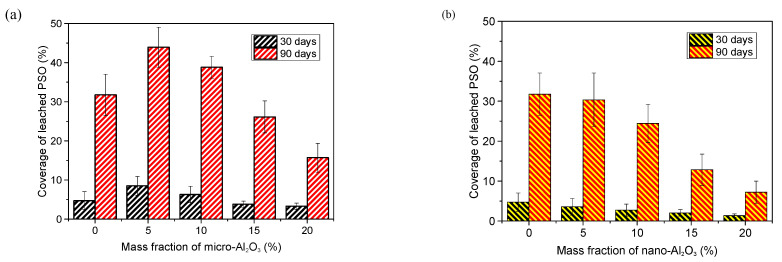
Effect of alumina on leached PSO: (**a**) Micro-alumina, (**b**) Nano-alumina.

The laser confocal morphology of the surface morphology of the coatings after exposure to air for 30 days and 90 days is shown in Figure 5. The black dots in the images are silicone oil droplets leached on the surface of the coatings. The coverage of leached PSO on coating obtained by image analysis is shown in Figure 6. Obviously, the coverage of leached PSO increases with exposure time. PSO leached decreases gradually with the increase of alumina content in the coating. When the content of alumina in the coatings is constant, the smaller the particle size of the powder is, the more difficult it is for PSO to be leaching on the surface of the coatings. Compared with the coating A-0, to which was not added alumina, all the coatings with nano-alumina leached more slowly and less PSO. The leaching behavior of PSO of the coatings with micro-alumina is, however, different from that of the coatings with nano-alumina. When the content of micro-alumina is 5% and 10%, it shows more leached PSO at the same exposure period. When the content of micro-alumina is more than 10%, though, the coverage of leached PSO is lower than that of the coating A-0. There is a dispersion limit of the powder in PDMS [19]. When the amount of the powder is lower, it can be dispersed by the resin and will not hinder the leaching of PSO. In addition, when the mass fraction is the same in the coating, the nano-alumina has a much high volume than the micro-alumina due to much lower density and less size., because the addition of alumina will effectively fill the microcracks and pores formed during the curing process of silicone and increase the resistance of silicone oil leaching. Alumina reinforced PSO/PDMS coating is the use of silicone oil leaching to enhance the anti-fouling and drag-reduction effect of the coatings. If the silicone oil is leaching too quickly, the antifouling period of the coatings will be shortened. Therefore, it is necessary to add an appropriate amount of alumina to control the leaching rate of the silicone oil.

### 3.3. Mechanical Properties

**Figure 7:**
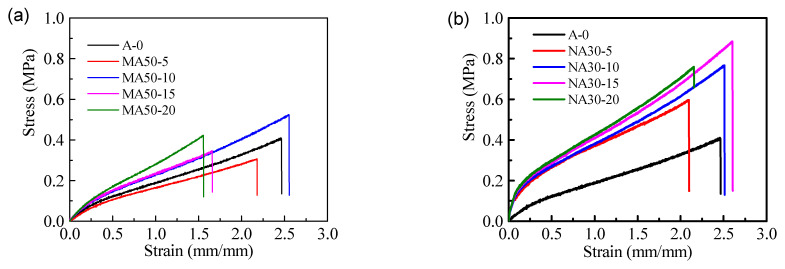
Stress-strain curves of tested coatings: (**a**) Micro-alumina, (**b**) Nano-alumina.

The addition of alumina can obviously improve the modulus of elasticity and shore hardness of the coatings, and the more alumina contained in the coatings, the greater the modulus of elasticity, as shown in Figure 7 and Table 2. 

Figure 7 shows that the elongation of micron alumina coatings is lower than that of nanometer alumina coatings. From the point of view of the particle size of alumina, the enhancement effect of nano-scale alumina on the mechanical properties of the coatings is greater than that of micron-level alumina. The mechanism of nano-alumina to enhance the mechanical properties of the coatings can be divided into two parts: First, nano-particles can effectively fill the microcracks and pores formed during the curing process of the coatings by their own small size. Second, the characteristics of the nano-particles’ own high hardness can enhance the mechanical properties of the coatings. The elastic modulus of the coatings is also related to the crosslinking density [27,28]. The swelling equilibrium method was used to determine the relative molecular weight (Mc) between the adjacent crosslinking points of the polymer, as shown in Table 3. 

The larger the Mc, the lower the cross-linking density [21]. When the amount of alumina in the coatings is the same, the Mc value of the nano-alumina coatings is much lower than that of the micro-alumina coatings. Therefore, the cross-linking density increases with the alumina content. The elastic modulus of MA50-5 is obviously lower than the normal value, which should be an operation error and should not be considered. The mechanical properties of similar PDMS coatings in some literature recently published were listed in Table 4. 

Comparing the results in Table 2 and Table 4, it is thus clear that the tensile strength, elongation at break, shore hardness and elastic modulus of pure PDMS coating can be greatly improved by adding reinforcing particles. The shore hardness and fracture elongation obtained in this study are similar to those in the literature [29,30,31]. The elastic modulus can reach 2–4 times that of the literature results. This is mainly due to the fact that the content (more than 5%, up to 20%) of alumina added in this study is much higher than that (about 1%) of particles added in the literature. 

**Figure 8:**
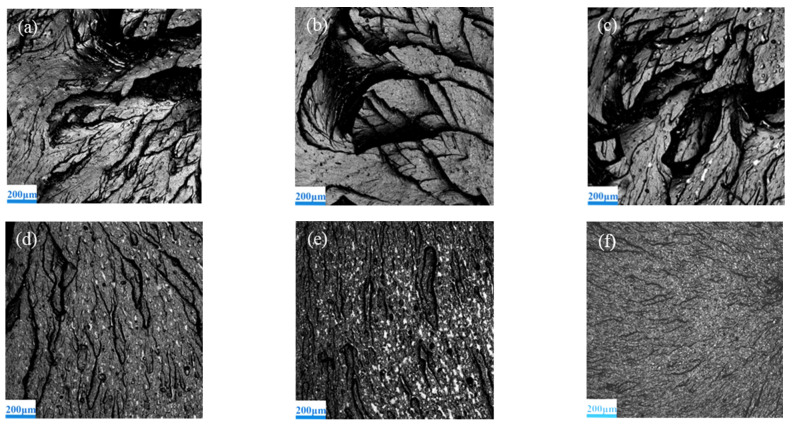
Tensile fracture morphology of alumina reinforced coatings: (**a**) MA50-5, (**b**) MA50-15, (**c**) MA50-20, (**d**) NA30-5, (**e**) NA30-15, (**f**) NA30-20.

The fracture morphology of the coatings was observed by CLSM, as shown in Figure 8. For micro-alumina coatings, as shown in Figure 8a–c, the fractures show large fluvial patterns, deep groove and cleavage fracture platforms. The fracture surface of micro-alumina coatings is step-like, with protrusion, voids and other defects. Because the micron powder cannot be uniformly distributed in the coatings, it will produce more defects, resulting in internal instability of the coatings [32,33]. Comparatively, as shown in Figure 8d–f, the fractures show small fluvial patterns, much shallower groove and much more and tiny cleavage fracture platforms. Because of the enhancement effect and dispersion effect of nanoparticles, the fracture micro-crack needs to bypass these particles before it can continue to expand. The fracture surface of nano-alumina coatings therefore changed, much smoother with increasing nano-alumina content. This is also the reason why the elongation of nano-alumina coatings is stronger than that of micro-alumina. 

### 3.4. Antifouling Performance

#### 3.4.1. Bacteria Attachment Performance

A bacteria attachment test was used to verify the antifouling performance of the coatings. With the addition and particle size of alumina as variables, different silicone topcoat samples were made. To directly find out the effect of alumina on the antifouling performance, the coatings with no silicone oil leaching on the sample platform for 7 days were selected in this paper. The number of bacterial colonies and the removal rate of fouling in each medium are shown in Table 5. CFU is the abbreviation of colony-forming units. Under the same flushing conditions, as the addition amount of alumina increases, the number of bacteria attached to the coatings will gradually increase. When the addition amount of alumina is constant, compared to the rinsed sample, the washed sample has less bacterial adhesion. In Table 5, as the addition amount and particle size of alumina increases, the fouling removal rate will gradually decrease. The bacteria removal rate decreases from 66.37% to 52.31%, which is related to the increase of the mechanical properties of the coatings.

#### 3.4.2. *Navicula Tenera* Attachment Performance

**Figure 9:**
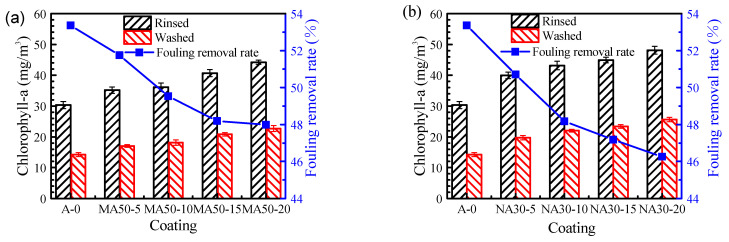
Effect of addition amount of alumina on the adhesion properties of *Navicula Tenera*: (**a**) Micro-alumina, (**b**) Nano-alumina.

A *Navicula Tenera* attachment test was used to verify the antifouling performance of the coatings. To directly discover the effect of alumina on the adhesion of *Navicula Tenera*, the coating with no silicone oil leaching on the sample platform for seven days was selected in this paper. We used an ultraviolet–visible spectrophotometer (Labtech UV-2000, Labtech Co., Ltd., Beijing, China) to measure the chlorophyll-a in the coatings; the more chlorophyll-a, the more *Navicula Tenera* attached to the coatings’ surface. The specific results are shown in Figure 9. Under the same flushing conditions, as the addition amount of alumina increases, the number of *Navicula Tenera* attached to the coatings will gradually increase, because the increase of alumina addition amount will enhance the mechanical properties of the coatings. As the addition amount and particle size of alumina increases, the *Navicula Tenera* removal rate will gradually decrease. The *Navicula Tenera* removal rate decreases from 66.37% to 52.31%.

#### 3.4.3. Influence of Elastic Modulus

**Figure 10:**
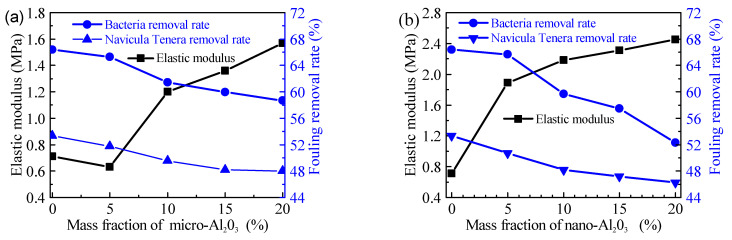
Elastic modulus and fouling removal rate of studied coatings: (**a**) Micro-alumina, (**b**) Nano-alumina.

According to the fracture theory, the fouling organism can be removed from the surface by peeling and plane shearing [31]. Because of the low elastic modulus of silicone resin, the fouling organisms leave the coatings surface mainly by peeling, and the energy required for peeling is much less than that of other methods. The energy required to peel off the fouling organism is equal to the adhesion energy of the fouling organism and the deformation energy of the coatings. Therefore, the better the elasticity of the coatings, the lower the energy required for peeling, and the more favorable it is for the fouling organisms to fall off from the surface of the coatings [29]. To alumina reinforced PSO/PDMS coatings, with the increase of its content, the fouling removal rate decreased slightly, because the addition of alumina will enhance the elastic modulus of the coatings, as shown in Figure 10. The elastic modulus of the coatings is inversely proportional to the fouling removal rate, which is consistent with the above findings. Alumina enhance the force required to peel off fouling organisms, thus reducing the fouling removal rate of the coatings. The decrease of fouling removal rate of nano-alumina is less than that of the micron one, which is also related to the enhancement effect of nano-powder. The nano-alumina can significantly enhance the mechanical properties of the coatings with its own high hardness.

### 3.5. Drag Reduction Performance

**Figure 11:**
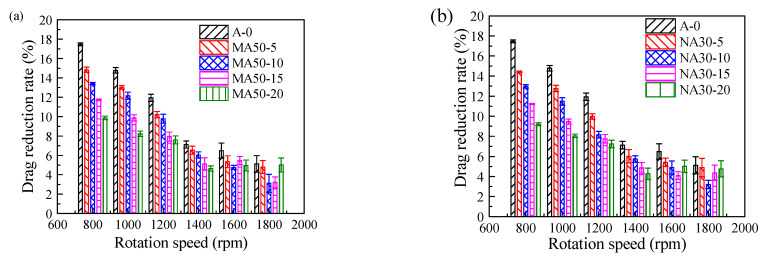
Drag reduction rate of studied coatings: (**a**) Micro-alumina, (**b**) Nano-alumina.

The drag reduction performance of the coatings was measured by using a rotating torque device. The torque (T) value represents the resistance to the rotation of the coatings in sea water. The greater the torque value, the greater the resistance. The relationship between the addition and particle size of alumina and the drag reduction rate (DR) is shown in Figure 11. Epoxy paint *HT*1 was chosen as the comparative coatings. This is the drag reduction rate Formula (1). Where *T_HT_*_1_ is the torque of comparative sample, $T_{X-Y}$ is the torque of the test coating X−Y.

(1) $$\mathrm{DR}\;=\frac{\left(T_{HT1}-T_{X-Y}\right)}{T_{HT1}}\times 100\%$$

**Figure 12:**
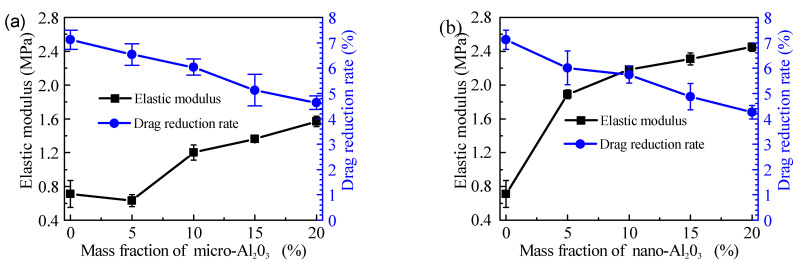
Elastic modulus and drag reduction rate of studied samples at 1400 rpm: (**a**) Micro-alumina, (**b**) Nano-alumina.

In Figure 11, under the condition of 800–1400 rpm, the increase of alumina content and the decrease of particle size will decrease the drag reduction performance of the coatings. Some studies have shown that flexible materials have an obvious drag reduction effect, because the elastic modulus of the material is low, which can release friction resistance through surface deformation [34,35]. In the alumina reinforced PSO/PDMS coatings, the addition of alumina will increase the elastic modulus of the coatings, thus reducing the buffering effect of the coatings. At 1400 rotational speed, the relationship between elastic modulus and drag reduction rate is shown in Figure 12. The results show that the elastic modulus is inversely proportional to the drag reduction rate, which proves that the elastic modulus is an important reason affecting the drag reduction rate.

**Figure 13:**
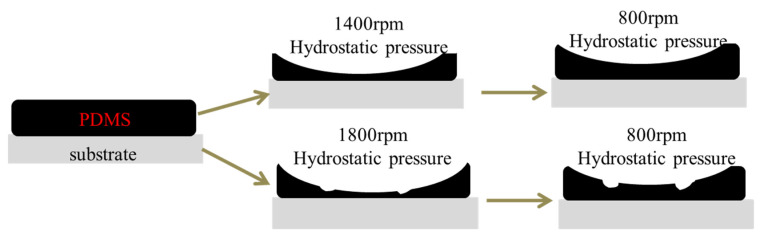
Schematic diagram of surface deformation of the coating.

When the rotational speed is higher than 1600 rpm, the change of drag reduction rate has nothing to do with the addition and particle size of alumina. This is because when the rotational speed does not exceed 1600 rpm, the elastic deformation of the coatings will counteract part of the resistance; that is, the coatings absorb and store this resistance. When the rotational speed exceeds 1600 rpm, the flow pressure exceeds the elastic limit of the coatings, which makes the coating unable to store energy, thus decreasing its drag reduction effect. The deformation diagram is shown in Figure 13.

### 3.6. Discussion

The above research results show that whether it is micron alumina or nano alumina, it can improve the elastic modulus, hardness and tensile strength of PDMS coating with the increase of alumina content. However, it reduces the antifouling, fouling-release and drag reduction properties of PDMS coatings to a certain extent. Despite all this, we cannot easily deny the possibility of alumina in improving fouling-release coatings. The reason is that the content range of alumina in this study may be too high, so it is necessary to further study the situation that the content of alumina is less than 5% in future investigation. In addition, there is a lack of results based on a long-term field panel test under actual marine conditions in this paper. After all, the experimental results obtained under the laboratory test conditions sometimes have a large deviation from the actual application field results. Moreover, the thickness of the coating is not considered in this paper; the influence of the thickness of PDMS coating on its antifouling and drag reduction properties and the mechanism are also worth studying in future. 

## 4. Conclusions

In this study, a series of alumina reinforced PSO/PDMS coatings were successfully manufactured to explore the effect of alumina addition amount and particle size on the performance of silicone coatings.

1. The water contact angle, surface energy and surface roughness of alumina reinforced PSO/PDMS coatings are 100–102°, 24–26 mJ/m^2^ and 0.14–0.21 μm, respectively. The content and size of alumina has little effect on the water contact angle, surface energy and roughness of the coating.
2. The leaching test of silicone oil shows that the increase of alumina content will hinder the leaching of PSO. The hinder effect of nano-alumina is stronger than that of micron alumina.
3. The addition of alumina can improve the mechanical properties of the coatings. With the increase of alumina content and the decrease of particle size, the elastic modulus increased from 0.71 MPa to 2.45 MPa, and the Shore hardness increased from 17.1 HA to 22.4 HA.
4. Introducing alumina reduces the antifouling properties of the coatings. This is related to the enhancement of mechanical properties. The removal rate of bacteria decreased from 66.37% to 52.31%, and removal rate of *Navicula Tenera* decreased from 53.36% to 46.26%.
5. At 800–1400 rpm, introducing alumina will weaken the drag reduction performance of the coatings, and it is found that the drag reduction rate is inversely proportional to the elastic modulus.
6. It is necessary to study the situation that the content of alumina is less than 5% and to carry out a long-term panel test in the sea in future investigation. Moreover, the influence of the thickness of PDMS coating on its antifouling and drag reduction properties and the mechanism are also worth studying in future.

## Figures and Tables

**Table 1:** Formulation of paints component A.

Sample	MA50-5 NA30-5	MA50-10 NA30-10	MA50-15 NA30-15	MA50-20 NA30-20
PDMS (g)	100.00	100.00	100.00	100.00
Additives and solvent (g)	25.00	25.00	25.00	25.00
PSO (g)	7.62	7.62	7.62	7.62
Micro/nano-alumina (g)	6.75	11.16	15.86	20.83

**Table 2:** Mechanical properties of Al_2_O_3_ reinforced PSO/PDMS coatings.

Coating	Elastic Modulus (MPa)	Fracture Strength (MPa)	Fracture Elongation (%)	Shore Hardness (HA)
A-0	0.71 ± 0.16	0.40 ± 0.06	246.55 ± 2.17	17.1 ± 0.21
MA50-5	0.63 ± 0.07	0.13 ± 0.04	218.13 ± 3.08	18.2 ± 0.31
MA50-10	1.20 ± 0.09	0.52 ± 0.08	255.34 ± 2.31	18.8 ± 0.17
MA50-15	1.36 ± 0.01	0.34 ± 0.05	165.95 ± 1.89	19.1 ± 0.25
MA50-20	1.57 ± 0.06	0.42 ± 0.03	155.80 ± 1.58	19.5 ± 0.16
NA30-5	1.89 ± 0.05	0.50 ± 0.05	209.74 ± 2.78	19.7 ± 0.21
NA30-10	2.18 ± 0.03	0.77 ± 0.06	251.11 ± 2.54	20.6 ± 0.37
NA30-15	2.31 ± 0.07	0.89 ± 0.08	260.28 ± 2.19	21.3 ± 0.25
NA30-20	2.45 ± 0.05	0.76 ± 0.05	215.02 ± 2.30	22.4 ± 0.31

**Table 3:** Mc values of Al_2_O_3_ reinforced coatings.

**Coating**	**A-0**	**MA50-5**	**MA50-10**	**MA50-15**	**MA50-20**
Mc	4648.52 ± 34.86	4179.76 ± 157.98	4010.85 ± 78.91	3994.62 ± 64.97	3905.74 ± 53.86
**Coating**		**NA30-5**	**NA30-10**	**NA30-15**	**NA30-20**
Mc		3557.16 ± 67.04	3328.34 ± 95.81	3179.15 ± 73.06	3084.30 ± 36.17

**Table 4:** Mechanical properties of PDMS coatings reinforced by different particles in literature.

Sample	Elastic Modulus (MPa)	Fracture Strength (MPa)	Fracture Elongation (%)	Shore Hardness (HA)	Literature
P0	0.09	0.05	39.00	6.57	[19]
M0	0.54 ± 0.021	0.53	273.00		[29]
M0.5	0.77 ± 0.026	0.55	241.00		[29]
M1	0.84 ± 0.047	0.59	189.00		[29]
Z30-1	0.44± 0.017			19.8 ± 0.37	[30]
Z50-1	0.38 ± 0.021			18.7 ± 0.51	[30]
Z100-1	0.32 ± 0.033			16.1 ± 0.17	[30]
Z100-4	0.47 ± 0.022			21.1 ± 0.41	[30]
Cu_2_O-1	0.73 ± 0.11			31 ± 0.55	[30]
FSiO_2_-1	0.68 ± 0.08			28.7 ± 0.47	[31]
Fe_2_O_3_-1	0.54 ± 0.02			26.1 ± 1.01	[31]
NSiO_2_-1	0.5 ± 0.08			23.3 ± 0.67	[31]
ZnO-1	0.45 ± 0.11			20.2 ± 0.74	[31]
CaCO_3_-1	0.37 ± 0.07			18.2 ± 0.33	[31]

**Table 5:** Bacterial colony concentration and removal rate.

Coating	Colony Concentration (×10^6^ CFU/mL)		Bacterial Removal Rate (%)
	Rinsed	Washed	
A-0	46 ± 1.31	15 ± 0.97	66.37
MA50-5	55 ± 1.43	19 ± 1.76	65.27
MA50-10	58 ± 0.94	22 ± 1.05	61.44
MA50-15	64 ± 1.47	25 ± 1.71	59.96
MA50-20	77 ± 1.79	32 ± 1.01	58.67
NA30-5	67 ± 0.91	23 ± 1.47	65.67
NA30-10	74 ± 1.45	31 ± 2.01	59.70
NA30-15	81 ± 1.21	34 ± 0.71	57.45
NA30-20	88 ± 2.11	43 ± 0.72	52.31

## Data Availability

Not applicable.
